# Hyperuricemia is associated with intermittent hand joint pain in a cross sectional study of elderly females: The AGES-Reykjavik Study

**DOI:** 10.1371/journal.pone.0221474

**Published:** 2019-08-23

**Authors:** Helgi Jonsson, Thor Aspelund, Gudny Eiriksdottir, Tamara B. Harris, Lenore J. Launer, Vilmundur Gudnason

**Affiliations:** 1 Landspitalinn University Hospital, Reykjavík, Iceland; 2 University of Iceland, Reykjavik, Iceland; 3 Icelandic Heart Association, Kopavogur, Iceland; 4 National Institute on Aging, Bethesda, MD, United States of America; University of Tasmania, AUSTRALIA

## Abstract

**Background:**

The debate whether "asymptomatic hyperuricemia" should be treated is still ongoing. The objective of this cross-sectional study was to analyze whether hyperuricema in the elderly is associated with joint pain.

**Methods and findings:**

Participants in the population-based AGES-Reykjavik Study (males 2195, females 2975, mean age 76(6)) answered standardized questions about joint pain. In addition they recorded intermittent hand joint pain by marking a diagram of the hand. In males, no association was found between hyperuricemia and pain. Females however, showed a positive association between hyperuricemia and joint pain at many sites. After adjustment for age, BMI and hand osteoarthritis however, only intermittent hand joint pain (OR 1.30(1.07–1.58), p = 0.008) and intermittent pain in ≥10 hand joints (OR 1.75(1.32–2.31), p<0.001) remained significant. The best model for describing the relationship between serum uric acid levels (SUA) and intermittent hand joint pain in ≥10 joints was non-linear with a cut-off at 372 μmol/L. The attributable surplus number of symptomatic females with SUA ≥372 μmol/L was approximately 2.0% of the study population for those reporting pain in ≥10 hand joints. Next after having severe hand osteoarthritis, SUA ≥372 was an independent predictive factor of intermittent pain in ≥10 hand joints. Intermittent hand joint pain was also an independent risk factor for worse general health description.

**Conclusion:**

Results from this population based study indicate that hyperuricemia in elderly females may be a rather frequent cause of intermittent hand joint pain, often in many joints. The most likely explanation relates to low-grade urate crystal induced inflammation. Our data do not allow for assessment of the severity of symptoms or whether they merit specific treatment, but intermittent hand joint pain was an independent predictor of worse general health. These findings may be an important contribution to the debate on whether hyperuricemia should be treated.

## Introduction

Hyperuricemia is part of a complex metabolic condition that is associated with age, gender, nutritional status including obesity and alcohol use, genetics, renal function and drugs, particularly diuretics. It´s prevalence appears to be rising globally. In the absence of gouty arthritis, hyperuricemia is considered to be asymptomatic as indicated by the commonly used term asymptomatic hyperuricemia[1,2].

In recent years, a number of studies have indicated that hyperuricemia may be deleterious to renal and cardiovascular health, but due to the complex interrelations between hyperuricemia and several other risk factors, there is still a lack of agreement whether it is the hyperuricemia itself that is harmful or it´s associated metabolic and life-style factors[3–6]. A recent review by Li et al. concludes that convincing evidence of a clear role of serum uric acid (SUA) level only exists for gout and nephrolithiasis[7]. There is also a lack of intervention studies in asymptomatic hyperuricemia, although some studies indicate a beneficial effect of uric acid lowering therapy on renal function both in patients with and without renal disease [8,9] and a reduced risk of developing hypertension in patients with type 2 diabetes [10]. In rheumatology, hyperuricemia and symptomatic gout appear to be a part of a spectrum, with crystal deposition and synovitis in joints sometimes evident even in the absence of clinical gout [11–13]. A recent NHANES study also indicates that hyperuricemia is associated with symptomatic knee osteoarthritis, most apparent in non-obese patients [14].

Due to the complexity of hyperuricemia and its association with other diseases and disease associated factors, large studies with extensive biochemical and health related information are necessary. The AGES-Reykjavik Study, a large population based study of elderly people in Reykjavik fulfills these criteria[15]. We have previously reported on osteoarthritis and pain in this population[16,17].

In the present study, we analyzed possible associations between serum uric acid levels and reported joint pain at different joint sites.

## Methods

The AGES-Reykjavik Study (Age, Gene/Environment Susceptibility—Reykjavik Study) is a population based study of elderly individuals from the 40 year long Reykjavik Study. Participants were aged between 66 and 96 and randomly recruited between 2002 and 2006. Written informed consent was obtained from all participants. Details of the investigations are described in the study´s baseline article[15]. The study is approved by the Icelandic National Bioethics Committee, (VSN: 00–063) and the Data Protection Authority.

The current study population is identical with our previous studies of osteoarthritis [16,17] with exclusion of individuals with evidence of inflammatory arthritis on hand photographs or having hand photographs that were unreadable with regard to hand osteoarthritis. This left a study group of 5170 subjects (2195 males and 2975 females, mean age 76(6)). Due to the marked gender differences in serum uric acid levels, males and females were analyzed separately. Hyperuricemia was defined as serum uric levels above the normal laboratory limits of 400 μmol/L in females and 480 μmol/L in males. Laboratory analyses were done using a Hitachi 912 analyzer (Roche Diagnostics) with reagents from Roche Diagnostics and following the manufacturer’s instructions. Reference values from The Nordic Reference Interval Project 2000 were used [18].

Study variables were acquired through laboratory tests, questionnaires and photographs, all taken at initial visit. Data about previous myocardial infarction were based on questionnaires, hospital records, the Icelandic Heart association registry and ECG. Similarly, previous coronary events also included CHD, PTCA and CABG.

### Questionnaire information

The pain questions were as follows:

Have you ever had pain, aching or stiffness in either knee, lasting for at least one month?

Have you ever had pain in or around either hip joint, including the buttock, groin or either side of the upper thigh, lasting at least one month?

Have you ever had pain lasting at least one month in the joints of your hands or wrist?

Have you ever had pain lasting for at least one month in your feet toes or ankles?

For each of the four questions above there was another starting by “In the past 12 months” followed by the same question.

In addition, participants filled out a diagram of both hands asking about sometimes joint pain and if yes, mark which particular joints (S1 and S2 Figs). The 20 alternatives were CMC1, IP1, DIP 2–5 and PIP 2–5 on both sides. In the article, those who gave a positive answer are referred to a having intermittent hand joint pain.

The question about general health was: In general, how would you rate your health? Excellent, Very good, Good, Fair or Poor. This variable was dichotomized into two parts as general health excellent, very good or good vs worse than good (fair or poor).

Education level was in four categories, corresponding to: primary school, secondary school, college and university. This variable was dichotomized into two parts by college education or higher.

The presence and severity of hand osteoarthritis was read from high quality digital photographs resulting in an aggregate score of 0–4 (HOASCORE) as previously described. Individuals with scores of 4 were classified as having severe hand OA[19].

The metabolic syndrome was defined according to the WHO definition criteria[20].

Data from the AGES study are available through collaboration (AGES_data_request@hjarta.is) under a data usage agreement with the IHA with an IRB approval in accordance with the informed consent.

### Statistics

Descriptive statistics are presented for participants with and without hyperuricemia according to laboratory normal values using t-test, chi square, and binary logistic regression. In the pain analysis age and BMI were used as covariates except for hand pain where the presence of severe hand OA was also used as a covariate.

On analysis of the relationship between SUA and pain the main outcome variables was joint pain treated as a binary variable: sometimes pain reported in 10 or more joints vs. the opposite. The outcome was analysed using binary logistic regression, with logit link, where the main predictor variable was serum uric acid. We used age (continuous), BMI (continuous), and severe hand OA (yes/no) as covariates.

We compared six types of models, each with a different representation of the main predictor variable: linear, spline, step, step with segment, segment, and hinge. Here linear representation refers to the classical logistic regression model where serum uric acid is linearly associated with the logit of pain. The spline model was fitted using generalized additive models (GAM) for logistic regression using a spline function[21]. The models of type: step, step with segment, segment, and hinge were fitted using threshold regression model estimation. Threshold regression models are a diverse set of non-regular regression models that all depend on change points or thresholds[22]. Standard error estimate for the cutoff value was generated using the bootstrap method with 1000 replications.

The models were compared using Nagelkerke’s R2, the AIC or the Akaike information criterion, and the AUC or the area under the receiver operating characteristics (ROC) curve. The contribution of the model variables to the prediction of pain was quantified by reporting the chi-square value of the likelihood ratio statistic from the multivariable logistic regression model.

The statistical analysis was performed using R version 3.4.2[23].

## Results

The characteristics of the study population in relation to the presence of hyperuricemia are shown in Table 1. The mean(SD) SUA in males was 382(91) and in females 332(93) μmol/L.

**Table 1:** Baseline characteristics in relation to hyperuricemia.

	MALES					FEMALES				
	Normouricemic		Hyperuricemic			Normouricemic		Hyperuricemic		
	(≤480μmol/L)		(>480μmol/L)			(≤400μmol/L)		(>400μmol/L)		
	n	mean(SD)	n	mean(SD)	p-value	n	mean(SD)	n	mean(SD)	p-value
**General**					t-test					t-test
Age	1908	76.4(5.3)	287	76.8(5.5)	0.31	2371	76.1(5.6)	604	77.5(7.7)	<0.001
Height(cm)	1906	175.5(6.1)	287	175.3(6.3)	0.52	2370	161.0(5.8)	603	160.3(5.7)	0.008
Weight(kg)	1907	82.1(12.9)	287	88.3(14.5)	<0.001	2370	69.1(12.8)	603	75.5(13.5)	<0.001
BMI(kg/m 2)	1906	26.6(3.7)	287	28.7(4.3)	<0.001	2370	26.6(4.6)	603	29.4(4.9)	<0.001
Abdominal circumference(cm)	1907	101.6(10.2)	287	107.2(11.1)	<0.001	2369	98.0(12.5)	604	99.4(12.9)	<0.001
**Laboratory serum concentrations**										
Fasting glucose(mmo/l)	1908	5.9(1.2)	287	6.1(1.1)	0.004	2371	5.6(1.1)	604	5.9(1.1)	<0.001
Cholesterol(mmol/l)	1908	5.2(1.1)	287	5.2(1.0)	0.40	2371	6.0(1.1)	604	5.9(1.2)	0.63
HDL(mmol/l)	1908	1.4(0.4)	287	1.3(0.4)	<0.001	2371	1.8(0.4)	604	1.6(0.4)	<0.001
hs-CRP(mg/l)	1908	3.6(6.7)	286	4.1(5.3)	0.17	2370	3.4(6.2)	604	4.6(5.2)	<0.001
Triglycerides(mmol/l)	1908	1,1(0.6)	287	1.5(0.9)	<0.001	2371	1.1(0.5)	604	1.5(0.8)	<0.001
Creatinin(μmol/l)	1908	97.9(24.3)	287	124.4(43.0)	<0.001	2371	78.1(20.5)	604	98.1(28.4)	<0.001
LDL(mmol/l)	1907	3.2(1.0)	287	3.3(0.9)	0.73	2370	3.7(1.0)	602	3.7(1.1)	0.89
HbA1c(%)	1763	5.7(0.6)	266	5.7(0.5)	0.22	2179	5.7(0.5)	552	5.8(0.5)	<0.001
**History**		percent	n	percent	x^2^	n	percent	n	percent	x^2^
Using antihypertensives	1908	58	287	91	<0.001	2371	57	604	92	<0.001
Past myocardial infarction	1895	17	285	22	0.07	2347	7	594	12	<0.001
Past coronary event	1895	32	285	39	0.03	2347	12	594	22	<0.001
Metabolic syndrome(WHO)	1895	21	285	48	<0.001	2350	15	363	39	<0.001
Severe hand OA (photo)	1908	8	287	9	0.81	2371	16	604	18	0.30
General health not good	1902	28	286	31	0.19	2368	34	603	42	<0.001
College education	1904	30	286	35	0.15	2366	24	603	19	0.006
Ever smoker	1906	72	287	70	0.62	2368	47	604	49	0.41
**Medication use**										
Thiazides	1908	18	287	49	<0.001	2371	29	604	63	<0.001
Loop diuretics	1693	8	277	26	<0.001	2162	5	592	20	<0.001
ACE-inhibitors	1693	17	277	31	<0.001	2162	10	592	18	<0.001
Hypoglycemics	1693	9	277	11	0.26	2162	4	592	7	0.001
Allopurinol	1908	5	287	6	0.56	2371	2	604	2	0.63
**Pain**										
Knee pain	1904	30	286	34	0.19	2368	40	603	43	0.16
Knee pain past 12 months	1907	19	287	22	0.30	2371	30	604	35	0.02
Hip pain	1902	18	285	22	0.14	2358	29	602	34	0.02
Hip pain past 12 months	1908	10	287	13	0.06	2371	18	604	22	0.03
Hand pain	1902	11	286	9	0.35	2366	26	603	29	0.15
Hand pain past 12 months	1908	6	287	5	0.89	2369	17	604	19	0.51
Foot pain	1895	17	285	16	1.00	2361	26	600	32	0.006
Foot pain past 12 months	1908	11	287	10	0.54	2371	20	604	26	0.002

UA levels showed (SUA) highly significant correlations with many of the variables in the table, associating positively with age, weight, BMI, abdominal circumference, fasting glucose, hs-CRP, triglycerides, creatinine, HbA1c, use of antihypertensive medications, the metabolic syndrome, history of previous cardiac events, worse general health and the use of thiazides, loop diuretics, ACE inhibitors and hypoglycemics. A negative association was seen with s-HDL(mmol/l) and education. No association was seen between SUA and severe hand osteoarthritis.

Hyperuricemia in males was not associated with reported pain. Unadjusted, females with hyperuricemia had significant association with reported pain at all four sites. On adjustment age and BMI were used, but based on previous findings of the association of hand osteoarthritis and hand pain [17], hand symptoms were also adjusted for the presence of severe hand osteoarthritis(HOA). The adjustment for severe HOA had negligible effect on association for other sites. After adjustment, only intermittent hand symptoms remained significant (Table 2). None of the other laboratory markers were associated with joint pain. The metabolic syndrome had an association with knee pain that vanished after adjustment for age, gender and BMI. Although hs-CRP was higher in hyperuricemic females, it showed no association with intermittent pain.

**Table 2:** The association between hyperuricemia and pain at different joint sites.

	**Males** (n = 2195, upper normal 480 μmol/L)					
	Unadjusted(Mantel-Haenszel)			Adjusted(Binary logistic regression with age, BMI* (and hand OA severity)**)		
	%pos	OR(95%CI)	p-value	OR(95%CI)	p-value	
Knee pain	31	1.20(0.92–1.56)	0.2	0.98(0.98–1.02)	0.9	*
Knee pain past 12months	20	1.18(0.87–1.60)	0.3	0.96(0.70–1.31)	0.8	*
Hip pain	18	1.27(0.94–1.72)	0.1	1.22(0.88–1.64)	0.3	*
Hip pain past 12months	10	1.44(0.99–2.09)	0.1	1.38(0.94–2.02)	0.1	*
Foot pain	17	0.99(0.71–1.40)	1.0	0.92(0.65–1.29)	0.6	*
Foot pain past 12months	11	0.87(0.57–1.31)	0.5	0.78(0.51–1.18)	0.2	*
Hand pain	11	0.79(0.51–1.23)	0.3	0.74(0.48–1.16)	0.2	**
hand pain past 12months	6	0.93(0.53–1.62)	0.8	0.86(0.49–1.51)	0.6	**
Intermittent hand joint pain	13	1.08(0.76–1.54)	0.7	1.03(0.71–1.48)	0.9	**
Intermittent hand joint pain ≥10 joints	2	1.25(0.52–3.02)	0.6	1.03(0.42–2.57)	0.9	**
	**Females** (n = 2975,upper normal 400 μmol/L)			Adjusted(Binary logistic regression with age, BMI* (and hand OA severity)**)		
	% pos	OR(95%CI)	p-value	OR(95%CI)	p-value	
Knee pain	41	1.14(0.95–1.37)	0.2	0.88(0.73–1.07)	0.2	*
Knee pain past 12months	31	1.27(1.05–1.53)	0.01	0.98(0.80–1.20)	0.8	*
Hip pain	30	1.13(1.03–1.51)	0.02	1.11(0.91–1.35)	0.3	*
Hip pain past 12months	19	1,28(1.03–1.59)	0.03	1.15(0.97–1.00)	0.2	*
Foot pain	27	1.32(1.09–1.60)	0.005	1.19(0.97–1.45)	0.1	*
Foot pain past 12months	21	1.40(1.13–1.72)	0.002	1.24(1.00–1.55)	0.05	*
Hand pain	26	1.16(0.95–1.42)	0.1	1.19(0.96–1.47)	0.1	**
Hand pain past 12months	18	1.08(0.86–1.36)	0.5	1.07(0.84–1.37)	0.6	**
Intermittent hand joint pain	38	1.37(1.14–1.64)	0.001	1.30(1.07–1.58)	0.008	**
Intermittent hand joint pain ≥10 joints	11	1.72(1.33–2.22)	<0.001	1.75(1.32–2.31)	<0.001	**

The association between uric acid levels and intermittent hand pain in females was consistent and clear, being highly significant for each and every of the 20 joints reported and strongest in those individuals reporting intermittent pain in many (≥10) hand joints.

### The relationship between SUA levels and intermittent hand pain

**Fig 1:**
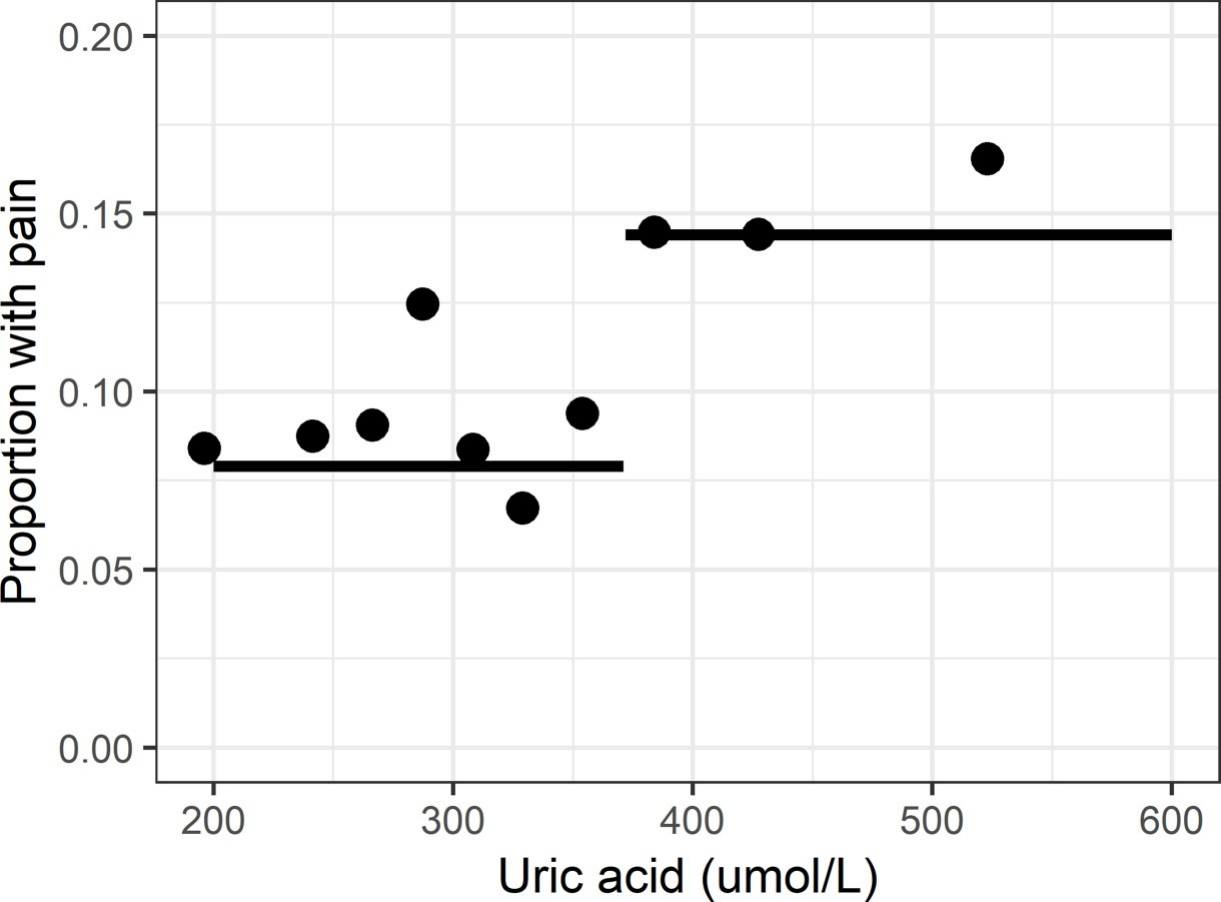
This figure where the dots represent deciles of SUA illustrates the best fitting threshold regression STEP model for the relationship between SUA levels and the proportion of females with intermittent hand pain in ≥10 hand joints by each decile. It also demonstrates the estimated cut-off at SUA ≥372 μmol/L.

We examined the relationship between SUA and intermittent hand joint pain in ≥10 hand joints by applying 6 different models of threshold regression. Uric acid was a predictor for pain in all 6 model types. The model with the highest R2, lowest AIC and highest AUC was the step model. This model was therefore chosen as the best fitting model (Fig 1).

The best fitting model for having intermittent pain in ≥10 joints was the non-linear step model indicating a cut-off for SUA at 372μmol/L (SE = 28). Below this level 8.9% had symptoms vs 15.8% with higher levels. This relationship is shown in Fig 1. The likelihood ratio statistics to quantify the strength of the association of the variables in the step model were: 0.3 for BMI (p-value 0.58); 16.9 for age (p-value <0.0001); 25.9 for Uric acid (as a threshold variable at the value 372) (p-value<0.0001); and 106.8 for severe hand OA (p-value<0.0001). Model comparison is shown in Table 3.

**Table 3:** Comparison of model fit statistics: Nagelkerke‘s Rs, Akaike Information Criterion (AIC), and the Area Under the Curve (AUC).

				AUC 95% CI	
Model	R^2^	AIC	AUC	Lower	Upper
Linear	0.086	1923	0.68	0.65	0.71
GAM	0.089	1923	0.68	0.65	0.71
Step	0.093	1913	0.69	0.66	0.72
Step Segment	0.093	1917	0.69	0.66	0.72
Hinge	0.086	1923	0.68	0.65	0.71
Segmented	0.088	1923	0.67	0.64	0.71

Attributable risk calculation indicated that the surplus number of females with SUA ≥372 μmol/L compared with those with SUA lower than 372μmol/L reporting intermittent pain in≥10 joints was 60 out of 2975 females or approximately 2% of the female study population. The surplus number was even higher for those reporting any intermittent pain (n = 85 or 2.9% of the study population).

### SUA and hand osteoarthritis as predictors of intermittent hand pain

**Fig 2:**
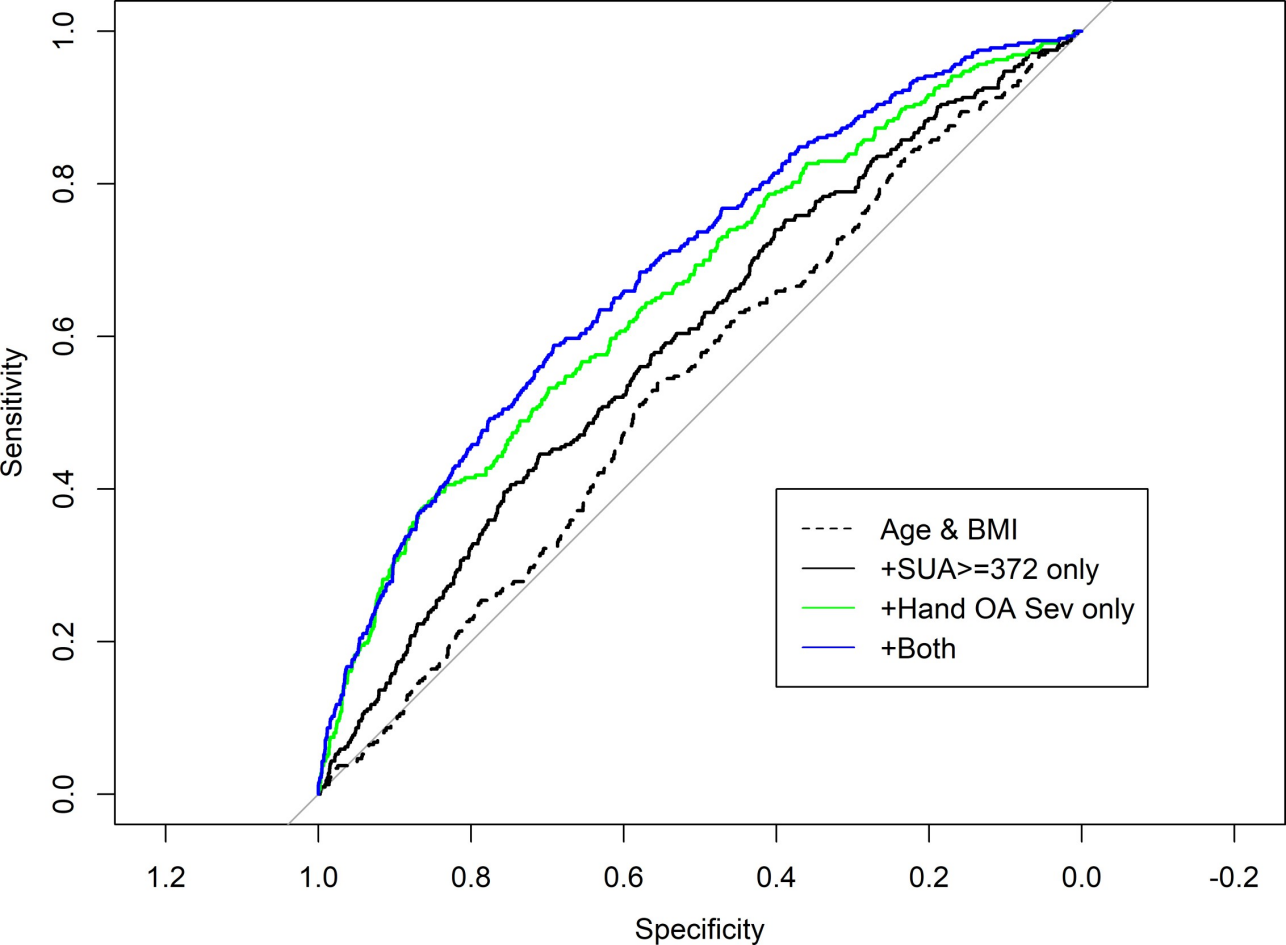
SUA levels and severe hand osteoarthritis as predictors of intermittent hand joint pain in ≥10 hand joints. The figure shows the ROC curves for 4 different models. Model 0 with just age and BMI, model 1 adding only SUA ≥372 μmol/L as a threshold variable, model 2 adding only severe hand OA, and model 3 adding both variables. The area under the ROC curves were respectively: 0.58, 0.60, 0.66, 0.69. The difference in area under the curve between model 2 and model 3 was statistically significant p = 0.005. In other words, adding SUA ≥372 μmol/L as a threshold variable to a model with age, BMI, and severe hand OA significantly improves the capacity of the model to diagnose pain.

In an attempt to estimate the relative contributions of hyperuricemia and hand osteoarthritis to intermittent hand pain in females we did a predictive power analysis for having intermittent pain in ≥10 hand joints. Adding joint pain at other sites to the model did not affect the results which are shown in Fig 2.

### Intermittent hand pain and reported general health

In an attempt to ascertain the severity and significance of intermittent hand joint pain we did an analysis of reported general health quality (dichotomized into excellent, very good and good vs fair or bad) and intermittent pain in females. Binary logistic regression revealed a highly significant negative associations between having intermittent pain in ≥10 hand joints and reported health quality. Regression analysis indicated an effect size (OR 3.0(2.4–3.8)) at least comparable to that of having pain lasting a month or more in feet (OR 2.5(2.1–3.0), knee (OR 1.9(1.6–2.2)) or hip (OR 2.1(1.8–2.5)

## Discussion

In this large population based study of elderly Icelanders, we found a highly significant association between hyperuricemia and intermittent hand joint pain in females, but no association in males. Even after adjustment for hand osteoarthritis severity, which is the strongest predictor of hand pain, SUA levels emerged as an independent predictive factor. These associations were particularly strong in those reporting intermittent pain in many hand joints. The association between SUA and intermittent pain in ≥10 hand joints was non-linear with a calculated cut-off at ≥372 μmol/L. Intermittent hand joint pain was in itself a highly significant predictor of worse general health with an effect at least comparable with having had foot, knee or hip pain lasting a month or more.

The most likely explanation for the episodic pain is inflammation due to urate crystal deposition. Dual energy CT imaging has demonstrated the occasional presence of urate deposits in joints and tendons, even in the absence of clinical gout[11]. A number of external factors such as usage, microtrauma and temperature as well as dehydration have been implicated in inducing gouty episodes[24]. Increased urate concentration appears to be the universal key factor in igniting the inflammatory response although other proteins and factors may act as promoters [25]. Interpreting the current findings, it appears that these metabolic and external circumstances may be present in the hand joints of elderly females. The non-linear association with a threshold level supports the concept of biochemical saturation reaching a threshold for crystallization and inflammatory episodes with pain. Regarding the current findings, it is important to realize that information about intermittent pain in our study was only available for the hand joints. It is likely that the traditional pain questions requiring pain episodes to last at least a month are insensitive in detecting hyperuricemia associated joint pain. The hand findings in the current study may thus apply to other joint sites. There have been a few studies that indicate that hyperuricemia is associated with foot and knee pain even in the absence of clinical gout[13,26], and the work of Stewart and Dalbeth on hyperuricemia related foot pain show considerable similarities with the current study findings[11,13]. In renal patients, hyperuricemia also seems to be an independent predictor of musculoskeletal symptoms[27].

Clinicians occasionally observe patients describing diffuse episodes of pain in multiple joints in the hands. Descriptions such as “hot or burning pain in my hands” are heard. It is interesting to speculate whether such episodes may be the hyperuricemia associated “sometimes pain” phenomenon we are describing in this study. Predisposing factors might include usage, microtrauma, exposure to cold and fluctuations in hydration status which is more common in the elderly [28]. Why the association between SUA and intermittent pain applies solely to females and not males is unexplained, but males report less pain at all sites.

Although uric acid induced inflammation may be the most logical explanation for intermittent pain in many hand joints, other explanations cannot be ruled out. The female population with SUA ≥372 μmol/L is older, less educated, heavier, has more evidence of a metabolic disorder, is using more antihypertensive medications, is more likely to have had cardiac events and reports worse general health compared to those with SUA <372 μmol/L. Any or all of these factors as well as possible alcohol consumption may contribute to hand pain through psychological mechanisms or increased pain sensitization. If this were the case however, one would expect a more general pain effect on other joint sites and also a higher prevalence of long lasting pain, not being limited to intermittent pain in the hands. These same factors may of course have bearing on the worse general health reported in the group with intermittent hand pain.

A limitation of this study is difficulty in finding other cohorts where these findings could be replicated. Unfortunately, the question about “sometimes pain” is non-traditional and we have not been able to locate a cohort where these results could be corroborated. Another limitation is the cross sectional nature of the data and our inability to assess the clinical significance of the hyperuricemia associated hand pain and whether it is of sufficient magnitude to merit specific interventions. Intermittent hand pain seems to be an independent predictor of general poor health, but once more the complexity of hyperuricemia associated conditions limits the scope for conclusions on this matter. The old age of the study population also limits conclusions with regard to the applicability of these results to other age categories, it may be limited to elderly females only, possible reasons being age-related changes in hand tissues and fluctuations in hydration status. The cut-off of SUA <372 μmol/L calculated in the current study may also vary between populations, depending on a number of other factors including obesity [14].

The current findings appear to indicate a hitherto undescribed clinical disease problem where 2–3% of the elderly female population suffer from intermittent hand pain that affects their general health. The condition is associated with a cut-off SUA level of ≥372 μmol/L raising questions of intervention in the form of uric acid lowering therapies. It is obvious that replication of the current findings is crucial as they could have extensive consequences with regard to the treatment of hyperuricemia.

## Supporting information

S1 Fig Pain diagram in Icelandic. (PDF)

S2 Fig Pain diagram in English. (PDF)
