# Supplementary material for: Hyperuricemia is associated with intermittent hand joint pain in a cross sectional study of elderly females: The AGES-Reykjavik Study
Source: PLoS One. 2019 Aug 23;14(8):e0221474. doi: 10.1371/journal.pone.0221474 (PMC6707588; doi:10.1371/journal.pone.0221474)
Supplement: S1 Fig — (PDF) [file pone.0221474.s001.pdf]

30670

Liðaverkir

Prófandi ☐☐☐☐☐☐☐☐☐☐

Ertu stundum með verki í liðum í höndum? ☐ Já ☐ Nei

Hvar finnur þú þá til? (Dæmi: "dofi þegar ég vakna", "mjög slæm þegar ég prjóna", "stirðir puttar í kulda") ______________________

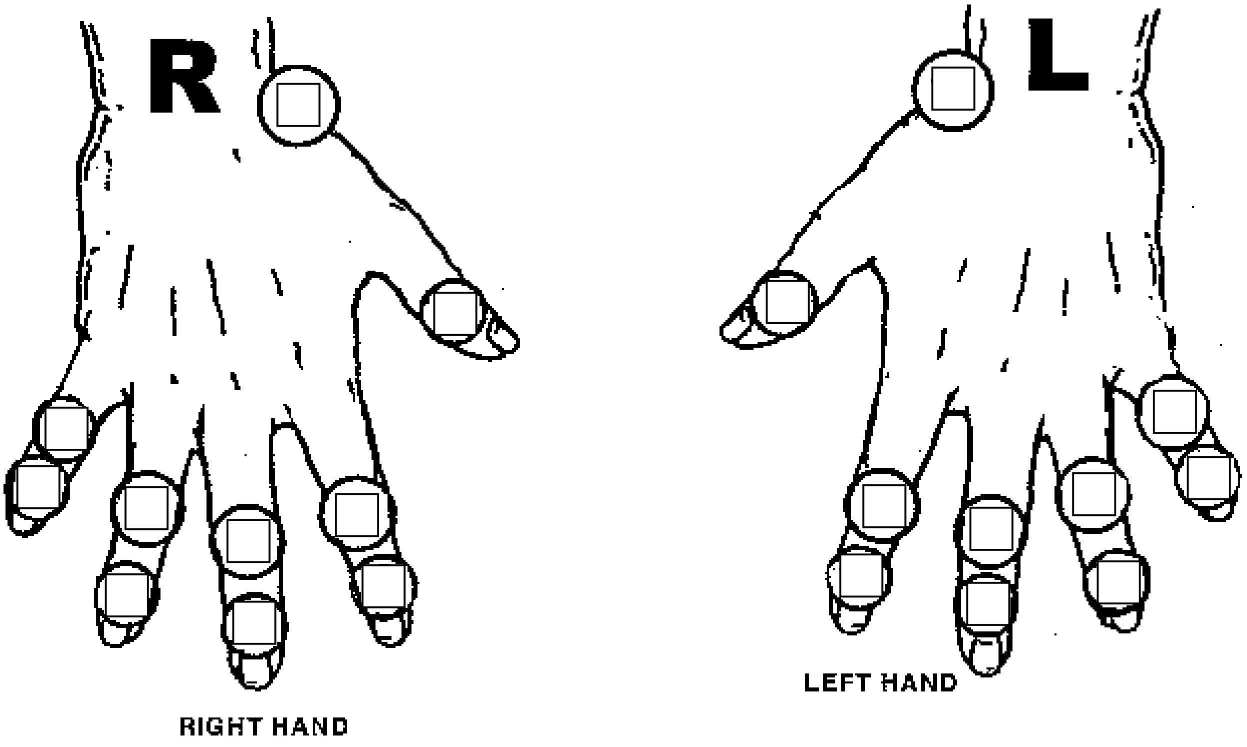

Hvað er merkt við marga liði á hægri höndinni?

☐ 0 ☐ 1 ☐ 2 ☐ 3 ☐ 4 ☐ 5

☐ 6 ☐ 7 ☐ 8 ☐ 9 ☐ 10

Hvað er merkt við marga liði á vinstri höndinni?

☐ 0 ☐ 1 ☐ 2 ☐ 3 ☐ 4 ☐ 5

☐ 6 ☐ 7 ☐ 8 ☐ 9 ☐ 10

Athugasemdir:
