# Supplementary material for: Hyperuricemia is associated with intermittent hand joint pain in a cross sectional study of elderly females: The AGES-Reykjavik Study
Source: PLoS One. 2019 Aug 23;14(8):e0221474. doi: 10.1371/journal.pone.0221474 (PMC6707588; doi:10.1371/journal.pone.0221474)
Supplement: S2 Fig — (PDF) [file pone.0221474.s002.pdf]

30670

Joint pain

Do you sometimes have pain in your hands? ☐ Yes No ☐

Where do you feel the pain? (mark the diagram)

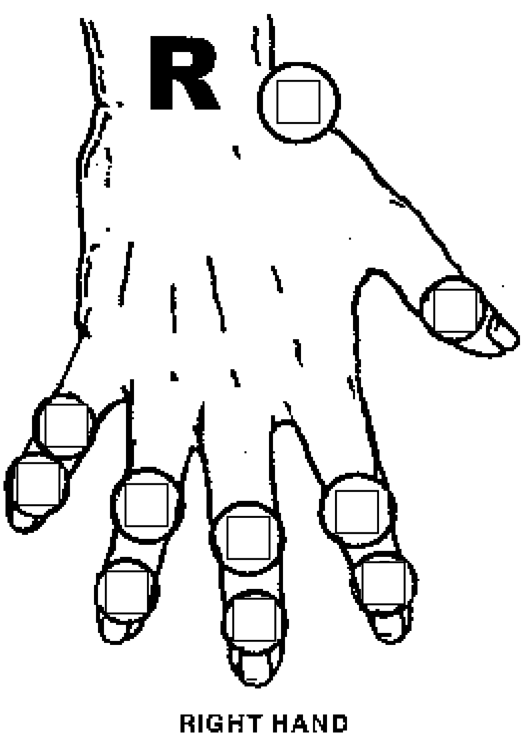

Comments:

Author's own translation from Icelandic.
